# Adaptation of Extended Reality Smart Glasses for Core Nursing Skill Training Among Undergraduate Nursing Students: Usability and Feasibility Study

**DOI:** 10.2196/24313

**Published:** 2021-03-02

**Authors:** Sun Kyung Kim, Youngho Lee, Hyoseok Yoon, Jongmyung Choi

**Affiliations:** 1 Department of Nursing Mokpo National University Muan Republic of Korea; 2 Department of Biomedicine Health & Life Convergence Sciences, BK21 Four Mokpo National University Muan Republic of Korea; 3 Department of Computer Engineering Mokpo National University Muan Republic of Korea; 4 Division of Computer Engineering Hanshin University Osan Republic of Korea

**Keywords:** nursing education, skill training, self-practice, smart glass, usability, feasibility

## Abstract

**Background:**

Skill training in nursing education has been highly dependent on self-training because of Korea’s high student-faculty ratio. Students tend to have a passive attitude in self-practice, and it is hard to expect effective learning outcomes with traditional checklist-dependent self-practice. Smart glasses have a high potential to assist nursing students with timely information, and a hands-free device does not interrupt performance.

**Objective:**

This study aimed to develop a smart glass–based nursing skill training program and evaluate its usability and feasibility for the implementation of self-practice.

**Methods:**

We conducted a usability and feasibility study with 30 undergraduate nursing students during a 2-hour open lab for self-practice of core nursing skills, wearing smart glasses for visualized guidance. The usability test was conducted using a 16-item self-reporting questionnaire and 7 open-ended questions. Learning satisfaction was assessed using a 7-item questionnaire. The number of practice sessions was recorded, and perceived competency in core nursing skills was measured before and after the intervention. At the final evaluation, performance accuracy and time consumed for completion were recorded.

**Results:**

Smart glass–assisted self-practice of nursing skills was perceived as helpful, convenient, and interesting. Participants reported improved recollection of sequences of skills, and perceived competency was significantly improved. Several issues were raised by participants regarding smart glasses, including small screen size, touch sensors, fogged lenses with masks, heaviness, and heat after a period of time.

**Conclusions:**

Smart glasses have the potential to assist self-practice, providing timely information at students’ own paces. Having both hands free from holding a device, participants reported the convenience of learning as they could practice and view the information simultaneously. Further revision correcting reported issues would improve the applicability of smart glasses in other areas of nursing education.

## Introduction

Advancements in life science and biotechnology have transformed the hospital environment, and the need for qualified health professionals has never been higher [1]. In most countries, nurses comprise the largest proportion of the hospital workforce; thus, nurse staffing with a sufficient number of skilled nurses is essential for quality care [2]. It is the responsibility of nursing schools at the undergraduate level to ensure quality care and patient safety with a well-organized curriculum. Practical training is as necessary as theoretical nursing education, and the importance of nursing skill training has been well recognized [3].

Acquisition of mastery in clinical nursing skills not only improves the overall quality of patient care, but also leads to a successful and confident nursing career [4]. Given the growing need for better assurance of practical ability, the Korean Accreditation Board of Nursing Education (KABONE) [5] identified 20 core nursing skills in which nursing students are expected to attain a good level of performance in their accreditation process [6]. Although performance exams have been widely implemented in other licensure examinations and for nurse licensure in other countries such as Canada [7], nursing education in Korea has fully relied on self-practice.

Regardless of the importance of skill training, challenges exist for running educational training programs in Korea. Schools lack the ability to accommodate necessary training because of high student-teacher ratios, so self-practice has been introduced as an alternative method for nursing skill training [8,9]. Considering the insufficient coaching and supervision, educational strategies are needed to improve the effectiveness and efficacy of this self-practice.

At present, students use a written checklist for self-practice, which provides text-based descriptions for each step. However, using these checklists without proper instruction from lecturers means that there is a high risk that students will misconduct their self-practice and repeat wrong performances. Prior studies have indicated that text alone is limited in delivering messages clearly when it contains complex issues [10,11]. The use of an image, which may be worth a thousand words [12], can make complex processes visible, which could effectively reduce the cognitive load involved in acquisition of skills [13]. Knowing that precise step-by-step implementation is essential, both comprehensive understanding and perfect memorization of each step would ensure excellent skill performance.

Visualization can be an effective solution, and its value for better learning engagement and active learning has been used recognized in education [14]. A benefit of visual representation is that a complicated process can be memorized more easily with graphically illustrated essential concepts. Currently, there has been a growing interest in using extended reality (XR) technology for training health care professionals [15]. With improvements in technology involving wearable devices, such as smart glasses, XR has been applied in many health care training programs [16-19]. The findings of these studies showed promising outcomes as effective alternatives to traditional educational programs. XR technology allows for new learning experiences via superimposition of holographic visualization on what users see in the real world.

Previous studies found passive attitudes among students participating in self-training programs, leading to a lack of competency in future nursing practice [10,20]. Furthermore, knowing that the training was insufficient, students lacked competency after completion of this unattended training, causing them anxiety and stress in nursing practice. XR technologies could be a solution, effectively assisting students’ self-training so that students are more likely to perceive that self-training is well structured and of high quality. In addition, the burden on faculty members to provide individual guidance can be alleviated because instant correction, where students reflect on timely information provided by smart glasses, is possible. Effective delivery of visualized education materials via an XR device could potentiate the learning experience without excessive consumption of educational manpower for supervision.

Along with visualization, timely information facilitates skill acquisition and completion. It is necessary to provide the experience of performing a true-to-life working process [21]. In addition, interacting with advanced technology could facilitate students’ motivation for self-practice. Previous studies showed that higher levels of attention and better learning engagement were achieved when implementing XR in education [22-24]. Using smart glasses enhances users’ engagement in performance [25]. Smart glasses improve the efficiency of practice, helping students master each skill with timely information without compromising performance. For complex skills, favorable consequences are expected to be higher, allowing students to experience a sense of accomplishment, completing exercises in a perfect manner, which would lead to improved competency in core nursing skills.

Smart glasses using augmented reality (AR) have previously been applied to support nursing care activities (eg, wound care management, mass casualty triage classification, and central line placement) [26]. These studies mostly focused on ease of obtaining knowledge and advanced features assisting the smart glasses’ performance. The positive implications of using smart glasses to assist in nursing activities were assured. The purpose of our study was to test the feasibility and usability of implementing a core nursing skill training program that combined visualization and XR technology for undergraduate nursing students. We hypothesized that a smart glass–based nursing skill training program would not only assist practice but also induce active engagement of students into self-training.

## Methods

### Design of Graphical Images for Screens

We developed an XR image guide training program for 2 core nursing skills, specifically, blood transfusion and intradermal injection administration. Of the 20 core nursing skills listed, these 2 skills were randomly chosen from those ranked high in difficulty level, classified by KABONE according to the procedures’ complexity. The numerous steps of these skills were split into several graphical images to be displayed on smart glasses. Each graphical image transposed to the smart glasses paralleled the text information in the original checklists. The contents of the XR image guide training program are shown in Table 1. The contents were developed and revised several times, considering conciseness and adequacy, which involved expert review by a team of 3 nursing faculty members (2 experts in fundamental nursing and 1 in nursing informatics) and user evaluation by nursing students. Students who participated in this user evaluation were asked whether the meaning was well delivered and could be recognized at a glance without misunderstanding the graphical image. The image was drawn as concisely as possible because of the small capacity of the screen within smart glasses. The conformity between the final version of the graphical image and text information in the checklists was reviewed by 2 professors who had more than 5 years of teaching experience in the fundamental nursing curriculum.

**Table 1:** Description of core nursing skills for smart glass–based self-practice.

Item	Steps, n	Core task	Necessary equipment and supplies
Blood transfusion	23	1. Preparing equipment and supplies. 2. Connecting blood product and injecting at right rate. 3. Noticing and warning for possible side effects. 4. Recording the nursing practice and patients’ conditions.	Number of items: 18 Manikin, blood product, alcohol swab, 3-way stopcock, gloves, IV^a^ pole, tray, watch with a second hand, stethoscope, sphygmomanometer, thermometer, kidney basin, recording paper, letter of consent, document needs sign, containers for general medical and damageable waste, and hand sanitizer.
Administration of intradermal injection	27	1. Preparing equipment and supplies. 2. Making diluted solution for AST.^b^ 3. Administering of intradermal injection 4. Reading the results of skin test.	Number of items: 12 Prescription, two 1-ml & 5-ml syringes, alcohol swab, manikin, vial, ampoule of normal saline, tray, recording paper, containers for general medical and damageable waste, and hand sanitizer.

^a^IV: intravenous.

^b^AST: antibiotic skin test.

### Preparation for User Study With a Smart Glass–Based Self-Training Program

A group of 5 students was given 2 hours of self-training and shared 2 Vuzix smart glasses. Students were encouraged to use the smart glasses at least once, with scheduled turns for the first use. According to KABONE, the estimated time to complete each skill was 10 minutes [5]. Before the beginning of their practice, brief instructions regarding how to operate the smart glasses (eg, content of the screen, how to find an item in the menu, turning to the next image) were provided, and individual students were given opportunities to wear the glasses and practice for about 5 minutes. After 2 hours of self-training, a performance test regarding accuracy and proficiency was conducted by an educator who had more than 5 years of experience in nursing education. During the test, the time for performance completion was measured by a research assistant.

**Figure 1:**
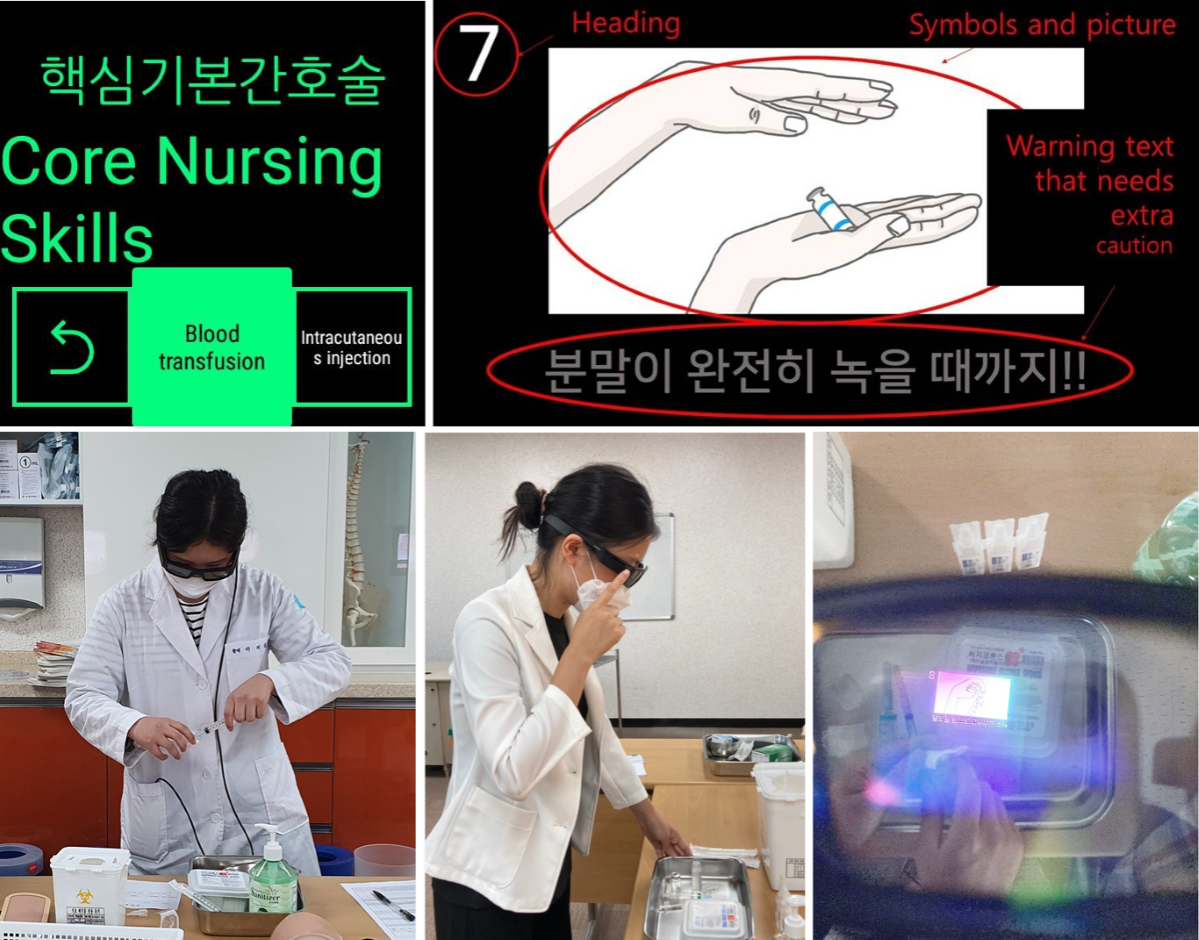
Composition and contents of smart glass displays.

Each image slide consists of 2 or 3 zones (Figure 1): (1) heading with sequence of the present step, (2) symbols and pictures representing the required performance in the present step, and (3) warning text that a step needs extra caution (when necessary).

The graphical images appeared in the order of actions following the sequence of the original text-based checklists. The 2 core skills of blood transfusion and intradermal injection administration were adapted to 23 and 27 screens, respectively (Figures S1 and S2 in Multimedia Appendix 1).

### Implementation of Our Smart Glass Application and Interface Design

The Vuzix Blade has a display only in the right eye, the display size is 480 × 853, and the shape of the display is a square. Vuzix’s appearance is similar to that of ordinary glasses, and it supports voice recognition and touchpads. It supports Bluetooth and Wi-Fi networks and has a camera attached to take photos and videos or engage in remote collaboration. It is a stand-alone device that weighs 93.6 g and does not require additional equipment. It has a screen saver, so users can use it like a transparent glass in normal times and turn on the display when they need information. The Vuzix Blade currently runs Android 5.1, which supports application programming interface (API) 22 for the target API. Developers can develop the software using Java or Kotlin using Android Studio. Differences from general Android programming include the voice recognition API, touch interface API, and heads-up display API for graphical user interface [27].

We set up our device as an always-on display. In general, smart glass displays are on-demand displays that turn off the screen after a certain period to save power. When information is only available for a short time upon request, job performance declines because of psychological pressure [28]. If the students had to touch the touchpad or call a voice command every time they requested information, it would waste their time, and they could become exhausted by simple repetitive tasks. Thus, we turned off the screen saver and kept the display on while the students practiced.

The Vuzix Blade allows user interface elements to be navigated with simple left/right/up/down navigation. The menu is expressed in a square shape at the bottom of the screen. Students can select a submenu by swiping and tapping the touchpad. When a student chooses a submenu, the corresponding image is displayed. We implemented a simple input method to reduce malfunctioning when students use the touchpad. When selecting a task in the list, they are only allowed to use the left/right swipe and one-finger tap. When flipping the slide, only left/right swipes were permitted. It was originally set up to swipe when moving to the upper menu, but we assigned a two-finger tap for moving to the upper menu.

### Usability Test

Seventeen items were used for the quantitative usability test. Items were developed based on previous studies in which relevant items were selected and revised to be aligned with the purpose and methodology of this study. The study participants reported perceived usefulness items and ease of use items on 5-point scales, from strongly disagree (1 point) to strongly agree (5 points).

### Learning Satisfaction

Level of satisfaction was assessed using 7 questions rated on a scale of 1 (strongly disagree) to 10 (strongly agree). Developed by Ji and Chung [29], questions were modified to fit the nursing education program best. With a maximum score of 70, a higher score indicates greater satisfaction with the education program.

### Nursing Competency

Levels of perceived competency on 2 core nursing skills (administration of intradermal injection and transfusion) were assessed using a 10-point Likert scale. Developed by Han, Cho, and Won [30], a higher score indicates a greater level of competency for each skill.

### Observation Data

During the 2-hour self-practice program, the overall number of practice attempts and number of practice attempts wearing smart glasses were observed and recorded by a research assistant. Developed by KABONE, a standardized checklist was used to measure the performance of 2 nursing skills (administration of intradermal injection and transfusion). The checklist consists of procedures of each skill, from preparing materials to writing nursing records after completion of skills. Scoring ranged from 0 to 100; a higher score indicates more accurate and precise performance without mistakes or omitted steps. At the nursing skill performance examination, individuals’ time spent on performance completion was measured and recorded by a research assistant.

### Qualitative Responses

Seven qualitative questionnaires were used to obtain comprehensive and detailed information about students’ experiences using smart glasses for core skill nursing training. The questionnaire included the following: (1) How did you find the smart glass–based training in general? (2) Was this program easy to use? Did you need additional instructions? (3) Was there any content causing confusion or difficulties? (4) Did you experience any difficulties while operating smart glasses? (5) Do you think it will be helpful for your future clinical practice? If so, how? (6) Would you make any recommendations that are needed to improve this training program? (7) If you have any other comments regarding this smart glass–based training program, feel free to add them.

### Ethical Considerations

The application of smart glass–based core nursing training for undergraduate nursing students was approved by the institutional review board (IRB no. MNUIRB-200326-BM-004-02) at a national university in Korea. Informed consent was obtained prior to obtaining the pretest data, and participants were told that they could stop participating anytime they wanted.

### Statistical Analysis

Quantitative statistical data analysis was conducted using SPSS (version 25.0; IBM Corp). Mean, SD, frequency, and percentage were calculated for the demographic data, observation data, usability, and learning satisfaction survey. Paired 2-tailed *t* tests were used to compare outcome measures preintervention and postintervention, and an independent *t* test was conducted to identify between-groups differences. Pearson correlation analysis was conducted to determine the association between variables. For the qualitative data, all responses were reviewed and coded to identify common themes that were frequently reported.

## Results

### Quantitative Findings

The mean age of the study participants was 22.70 years, and 63% (19/30) were female. Approximately two-thirds of participants (22/30, 73%) reported possessing a moderate level of competency in core nursing skills, and approximately 1 in 10 (3/28, 11%) had previous experience with AR (Table 2).

**Table 2:** Demographic characteristics of study participants (N=30).

Characteristics		Value	
Age (years), mean (SD)		22.70 (1.39)	
**Gender, n (%)**			
	Male		11 (37)
	Female		19 (63)
**Academic grade, n (%)**			
	Good		4 (13)
	Fair		18 (60)
	Poor		8 (27)
**Satisfaction with clinical placement, n (%)**			
	High		21 (70)
	Moderate		9 (30)
	Low		0 (0)
**Core nursing skill competency, n (%)**			
	Good		8 (27)
	Fair		22 (73)
	Poor		0 (0)
**Previous experience with augmented reality, n (%)**			
	Yes		3 (11)
	No		25 (89)

Participants were given 2 hours of open lab for self-practice of 2 core nursing skills. The number of practice attempts varied between 5 and 9. Full usage of the smart glasses during the self-practice open lab was observed and recorded. Participants used smart glasses in their practice as little as 2 and as many as 6 times (Table 3).

**Table 3:** Number of practice attempts and smart glass use.

Categories			Range		Mean (SD)
**Practice attempts (total), n**					
	Blood transfusion	2-6		3.30 (0.952)	
	Administration of intradermal injection	2-5		3.73 (0.944)	
	Total	5-9		7.03 (1.25)	
**Practice attempts wearing smart glasses, n**					
	Blood transfusion	0-3		1.83 (0.747)	
	Administration of intradermal injection	0-3		1.70 (0.651)	
	Total	2-6		3.53 (0.973)	

Regarding self-reported usability of the smart glass–based self-practice program, the highest score was obtained for question 11 (perceived interest) with a mean of 9.50 (SD 0.86). Question 5 (screen resolution) scored the lowest with a mean of 7.20 (SD 2.02). The degree of difficulties experienced with devices was rated with a mean of 3.83 (SD 2.73) (Table 4).

**Table 4:** Results of 16-item usability test (N=30).

Item		Range	Usability, mean (SD)
**Ease of use**			
	1. How convenient do you think the smart glass–based core nursing education program is?	3-10	8.10 (1.58)
	2. Was the initial education regarding the device and usage appropriate?	5-10	8.77 (1.46)
	3. Was the text information presented on the screen easy to read?	3-10	7.27 (2.26)
	4. Was the picture information presented on the screen clearly understood?	4-10	8.17 (1.90)
	5. Was the resolution of the screen good?	4-10	7.20 (2.02)
	6. Did you have any difficulties because of errors that occurred during the performance?	1-10	3.83 (2.73)
	7. Was the progression speed adequate?	5-10	8.63 (1.22)
	8. Was the location of the information on the smart glass appropriate? Consistent? Easy to see?	3-10	8.53 (1.85)
	9. Was it convenient to operate the smart glass?	6-10	8.40 (1.48)
**Usefulness**			
	10. Did the pictures and text information shown help you perform core basic nursing skills?	6-10	9.07 (1.05)
	11. Was this type of educational program interesting?	7-10	9.50 (0.86)
	12. Did you expect better scores using the smart glass training program?	6-10	8.90 (1.21)
	13. Did you have a better understanding of core basic nursing techniques using augmented reality?	5-10	8.77 (1.48)
	14. Would you recommend the smart glass–based core nursing education program to other friends?	3-10	8.70 (2.00)
	15. Do you think smart glass core nursing education will be useful in clinical practice in the future?	4-10	8.77 (1.61)
	16. Are you willing to use a smart glass for other core nursing skills in the future?	5-10	8.87 (1.57)

The overall score for learning satisfaction was 9.00 (SD 0.72). The participants gave the highest rating to “It was an interesting learning experience” (mean 9.60, SD 0.68) and the lowest rating to “It was more effective than lecturer-based education” (mean 7.43, SD 1.81) (Table 5).

**Table 5:** Results of 7-item learning satisfaction score (N=30).

Item	Range	Mean (SD)
1. It was an interesting learning experience.	8-10	9.60 (0.68)
2. Educational goals of this program were well-achieved.	7-10	9.30 (0.88)
3. It was a meaningful learning experience.	8-10	9.47 (0.73)
4. It was more effective than lecturer-based education.	3-10	7.43 (1.81)
5. I actively engaged in learning.	7-10	9.27 (0.91)
6. I felt satisfied with the educational program overall.	7-10	9.23 (0.90)
7. I hope to use this educational program for other subjects.	3-10	8.73 (1.46)
Learning satisfaction score (total)	N/A^a^	9.00 (0.72)

^a^N/A: not applicable.

Paired 2-tailed *t* tests were used to analyze differences between preintervention and postintervention competency of students on 2 nursing skills. Statistically significant improvement was achieved in both the skills after intervention (*P*<.001) as mean scores increased from 7.23 (SD 1.17) to 8.90 (SD 0.85) and 6.17 (SD 1.64) to 8.50 (SD 0.97), respectively (Table 6).

**Table 6:** Perceived competency in nursing skills before and after smart glass–based self-practice (N=30).

Item, time		Mean (SD)	*t* test (*df*)	*P* value
**Administration of intradermal injection**			−6.53 (29)	<.001
	Pre	7.23 (1.17)		
	Post	8.90 (0.85)		
**Blood transfusion**			−7.00 (29)	<.001
	Pre	6.17 (1.64)		
	Post	8.50 (0.97)		

The Pearson correlation test was conducted and identified statistically significant negative correlations between the number of practice attempts wearing smart glasses and performance time (*r*=−0.666, *P*<.001). There was a statistically significant positive correlation between the number of practice attempts wearing smart glasses and learning satisfaction (*r*=0.404, *P*=.03) (Table S1 in Multimedia Appendix 1). An independent *t* test revealed statistically significant gender differences for usability scores (ease of use, *P*=.049) and previous experience with devices (ease of use, *P*=.02; usefulness, *P*=.002) (Table S2 in Multimedia Appendix 1).

### Qualitative Findings

#### Overall Experience of the Smart Glass–Based Skill Training Program

In general, the majority of students found smart glass–based skill training interesting (13/30, 43%) and convenient (8/30, 27%). About one in three participants (8/30, 27%) did not find significant benefits of using smart glasses for self-practice, and one participant reported, “It was interesting at first, but previous text based learning fits better for me.” Resistance to learning new technology was revealed, with participants saying, “I think I had to make more effort to learn about the devices.”

With regard to smart glasses, a large number of participants reported some degree of discomfort. There were touch sensor–related issues (9/30, 30%), specifically, “The touch sensor was too sensitive.” Others complained about the smart glass screen, and participants said, “Small sized text and low resolution caused eyestrain.”

Generally, participants responded that this smart glass–based self-training has educational benefits. Some participants found increased engagement in learning with new technology, saying “I was fascinated by the smart glasses and practice became more interesting using it.” Participants responded to the effectiveness of visualized information, improving their memory of educational content (n=7). Moreover, timely provision of information was found to have significant positive benefits (n=8): “Assisted by timely provided information, accurate and seamless practice was ensured using smart glasses.”

#### Perceived Easiness

Overall, the participants considered smart glass–based training to be very intuitive. Two out of three (20/30, 67%) reported immediate adaptation to smart glasses, with one participant saying “It was quite straightforward, I figured out how it works right away.” One-third of participants expressed the need for additional instructions, with one saying “I got confused with device, especially the touch pad on the glasses did not make sense for me.”

#### Recommendations

Several participants provided feedback regarding areas that needed further improvement. In terms of the smart glasses, participants raised the following issues: (1) touch sensor not working properly while wearing latex gloves, (2) glasses easily fogging while wearing a mask, (3) pain of double-layering glasses for people with poor eyesight, and (4) discomfort due to heavy weight and heat after a period of time (about 15 minutes). Regarding the training program content, participants reported tediousness of the simple text and image, saying “I expected something more entertaining like games.”

## Discussion

### Principal Findings

We explored the perceived usability and feasibility of smart glass–based self-practice among undergraduate nursing students. In general, the findings indicate that the participants had a greater degree of interest in this new device. Although some participants showed resistance to learning about the device, most students were pleased with having new educational methods to assist in their self-practice. This is closely linked to the characteristics of the study population. Recent advances in computing technology have transformed education, and the current generation is accustomed to this continuous change [26].

The findings of this study revealed the positive effects of smart glasses on engaging students in self-practice. Like self-practice, where an active learning attitude is essential, smart glasses could certainly provide a learner-centered education platform, allowing learning at an individual’s own pace without restrictions of time and supervisors [31]. This learner-centered approach enables students to make the best use of learning materials. As a matter of fact, participants who showed high levels of learning satisfaction used the smart glasses more frequently. This indicates that active participation is closely linked to the attractiveness of education strategies, which would eventually lead to learning satisfaction. Knowing that implementation of smart glasses induced students’ interest in skill practice, thoughtful consideration is required for nursing faculty members, who should make efforts to identify applicable areas where this technology can be used.

The findings of this study indicate greater improvement in perceived competency of performing blood transfusions. One possible explanation is variability in individual skills. Blood transfusion requires not only skillful performance but also complex procedures that involve multiple confirmation process (eg, physicians’ orders, blood continuants, lab examination results, monitoring patients for side effects) [32] that frequently cause low levels of competency among undergraduate nursing students. Thus, by following guidance provided via smart glasses, students may find the tasks easier to complete. In addition, students would be better equipped with more complete knowledge regarding the step-by-step processes of blood transfusion, leading to increased competency.

In terms of number of uses, there was a positive correlation with learning satisfaction and a negative correlation with time consumed on performance completion. In addition, participants responded to educational benefits, as a timely graphical image assisted in improving their memory of the correct sequences. This suggests that the use of smart glasses has great potential to boost students’ abilities for task completion. This is in line with previous studies on AR smart glasses, which assist health professionals in enabling simultaneous performance of multiple tasks [28,33]. One possible explanation is better cognitive performance due to greater efficacy in information confirmation provided via the glass screen and easy recollection of graphic-based guidance. Similarly, fast-tracking (shortening) the link between knowledge acquisition and actual practice or even tightly coupling them concurrently could expand individuals’ existing abilities. Although usage frequency is closely related to students’ preferences for this kind of learning, cumulative evidence would easily induce active engagement.

Several responses involved feedback that needs further consideration for the use of smart glasses to practice skills in nursing education, including low resolution, lack of visibility due to small-sized text, light smudging, perceived heaviness, severe condensation when wearing masks, and pain and discomfort for users wearing eyeglasses. Smart glasses have been considered for clinical implementation such as medical remote collaboration; thus, this is crucial information for future studies. Tasks are (1) determining the optimal size of text for users with poor eyesight, (2) identifying colors that cause light smudging, and (3) comparing and selecting smart glasses that are less likely to cause these issues. For example, the recently developed Google Glass may overcome some of these drawbacks with its advanced display, customizable hard case (lighter version without lenses or version with a thicker and solid frame), and lightweight form factor [34].

Findings of learning satisfaction outcomes revealed relatively lower scores on the item: “It was more effective than lecturer-based education.” This indicates a limitation of the smart glass–based self-practice program. Although students’ practice was assisted by the smart glasses, it could not sufficiently replace lecturer-based education. This implies the need for additional strategies to meet the educational needs of nursing skill training. Integration of prior educational strategies could effectively reinforce the current version of smart glass–based education.

The diverse features of smart glasses would more effectively replace previous strategies used for self-practice in nursing education. First, self-practice with video recording with self-feedback or peer feedback, the effectiveness of which was well established in a previous review study [35], could well be administered using smart glasses in a simpler and more convenient manner. Second, a demonstration from the lecturer can also be delivered via a smart glass guidance system. Inserting recorded videos or a series of photos in a GIF for demonstration could well guide students’ acquisition of nursing skills. Lastly, consideration for aligning procedural steps with multimodal feedback, such as notification timer, sound, and vibration effect, is needed. There is evidence that gamification contributes to teaching and learning in nursing education [36], and these features have a high potential to immerse learners in self-training.

We conducted further statistical analysis (Tables S1 and S2 in Multimedia Appendix 1) to identify the potential influence of various user characteristics such as age, gender, and previous experience with devices. Interestingly, female participants and participants with no previous experience with AR devices reported better usability of the current smart glass–based training program. Previously, males were believed to be more willing to use and adapt more quickly to new technologies [37], while other studies observed no gender differences [38,39]. The findings of this study partially align with those of a recent study by Drin, Alamaki, and Soumala [40], which reported greater interest among females toward new technology. Novelty effects might be related to the lower usability scores of participants with previous experience; this may be related to prior experience negatively affecting attitudes toward the present experience [41]. Another possible explanation is that students’ perceptions of the current smart glass–based training program might be influenced by their perception of the program itself. Since this study was intended to promote self-practice for nursing skills, a passive attitude toward training was reflected. Regardless of gender, age, and previous device experience, students’ willingness and active attitude result in greater educational benefits. Further investigation regarding influencing factors on the user’s perception toward smart glasses and their applications for education would offer a more comprehensive understanding for future developers.

### Limitations

This study was not without limitations. First, although this was a pilot study focusing on usability and feasibility, the small sample size restricted the interpretation of some of the results. In addition, it is not possible to fully elucidate the effectiveness of the smart glass–based training program. Examining usability and feasibility, we did not thoroughly compare the effectiveness of the smart glass–based training program to other existing training programs that are prevalent in nursing education (eg, smartphone video recording of self-practice). Given the finding from this study that smart glasses can be a useful education strategy, more thoroughly demonstrating the effectiveness with future research would encourage faculties to actively incorporate such devices into their education plans. Lastly, it is questionable whether the Vuzix Blade is the best device for nursing skills training, as new smart glasses are continuously released in this growing market. Thus, future research using a variety of smart glasses with differing specifications that reflect factors that caused discomfort and inconvenience in this study would offer valuable information for educators considering the use of smart glasses. Employing and comparing various AR presentation types (eg, 3D content, data visualization, virtual characters) and AR augmentation techniques (eg, multimodal, physical feedback, sound augmentation) are worthy of further investigation to elucidate optimal smart glasses–based practices.

### Conclusion

The findings of this study suggest the use of smart glasses was a useful educational strategy for assisting self-practice of skills in nursing education. Given the benefits of timely information and hands-free operation (hands free from holding a device), participants reported positive experiences in general, including a high level of interest and appreciation for the convenience of this training program. Participants who had favorable views of this technology-enhanced education were more likely to report greater learning satisfaction, which shows great potential in transforming a previously passive attitude to an active one. Future revision reflecting the feedback from this study would effectively foster a high level of skill competency among undergraduate nursing students, engaging students in active learning and reducing the burden on faculty members.

## Appendix

Multimedia Appendix 1 Snapshots of the image guide for intradermal injection, snapshots of the image guide for blood transfusion, Pearson correlation analysis among study variables, and difference of usability score (ease of use and usefulness) by gender and previous experience of augmented reality.

## Abbreviations

API: application programming interface
AR: augmented reality
KABONE: Korean Accreditation Board of Nursing Education
NRF: National Research Foundation of Korea
XR: extended reality
