# Supplementary material for: Adaptation of Extended Reality Smart Glasses for Core Nursing Skill Training Among Undergraduate Nursing Students: Usability and Feasibility Study
Source: J Med Internet Res. 2021 Mar 2;23(3):e24313. doi: 10.2196/24313 (PMC7967227; doi:10.2196/24313)
Supplement: Multimedia Appendix 1 [file jmir_v23i3e24313_app1.docx]

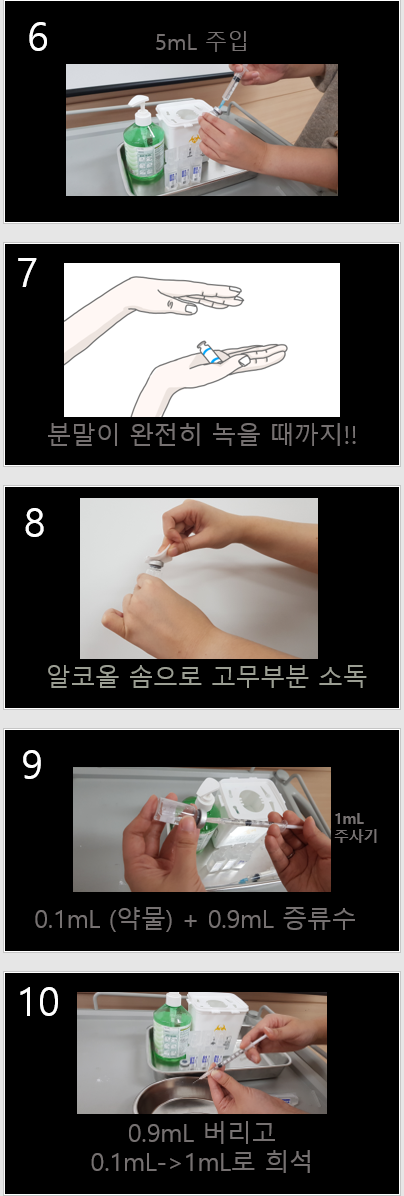

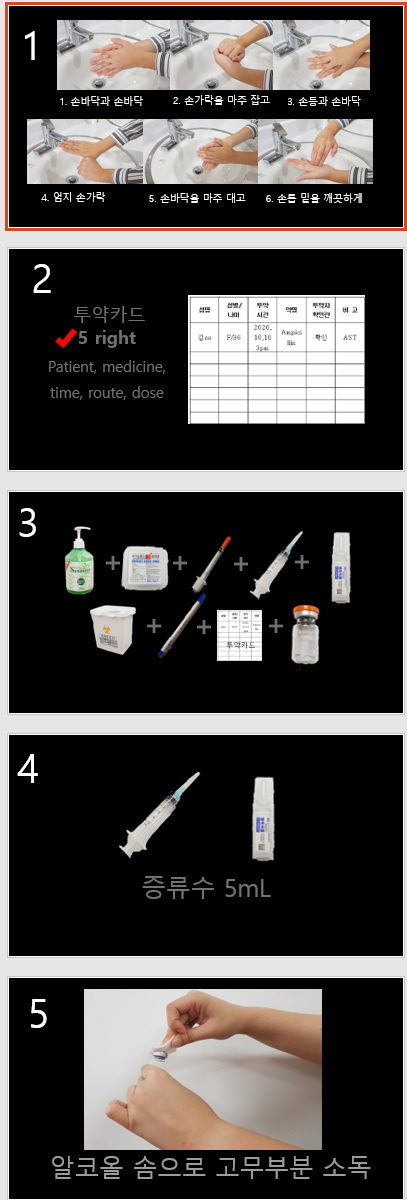


Figure S1. Snapshots of the image guide for intradermal injection


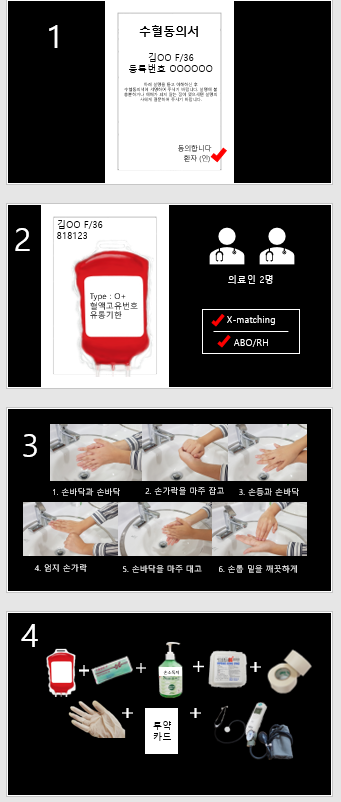

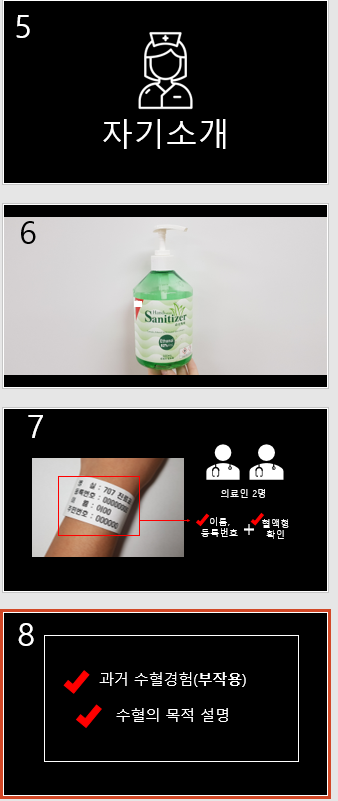


Figure S2. Snapshots of the image guide for blood transfusion

Table S1. Pearson’s correlation analysis among study variables

|  | 1 | 2 | 3 | 4 | 5 | 6 | 7 | 8 | 9 | 10 | 11 |
| --- | --- | --- | --- | --- | --- | --- | --- | --- | --- | --- | --- |
| 1. Age | 1 |  |  |  |  |  |  |  |  |  |  |
| 2. Number of practice attempts (total) | -.034 | 1 |  |  |  |  |  |  |  |  |  |
| 3. Number of practice attempts wearing smart glasses | .020 | .241 | 1 |  |  |  |  |  |  |  |  |
| 4. Performance completion time | .085 | -.391^*^ | -.666^**^ | 1 |  |  |  |  |  |  |  |
| 5. Performance test score | .293 | .274 | .031 | -.343 | 1 |  |  |  |  |  |  |
| 6. Learning satisfaction | -.003 | .313 | .404^*^ | -.422^*^ | .021 | 1 |  |  |  |  |  |
| 7. Usability score (ease of use) | -.303 | -.056 | .150 | -.015 | .064 | .368* | 1 |  |  |  |  |
| 8. Usability score (usefulness) | -.185 | .202 | .382* | -.386* | .131 | .639** | .750** | 1 |  |  |  |
| 9. Level of competency for core nursing skills (prior to training) | .572** | -.263 | .215 | -.053 | .115 | -.035 | -.264 | -.127 | 1 |  |  |
| 10. Changed competency (intradermal injection) | -.266 | .165 | .237 | -.213 | -.009 | .244 | .159 | .236 | -.238 | 1 |  |
| 11. Changed competency (transfusion) | -.230 | .086 | .304 | -.200 | .010 | .124 | .052 | .126 | -.238 | .896^**^ | 1 |

**P* < .05, ***P* < .001

Table S2. Difference of usability score (ease of use and usefulness) by gender and previous experience of augmented reality

| Usability | Characteristics | Categories | N | Mean (SD) | t | p |
| --- | --- | --- | --- | --- | --- | --- |
| Ease of use | Gender | Female | 19 | 8.37 (0.70) | 2.17 | .049 |
|  |  | Male | 11 | 7.43 (1.32) |  |  |
|  | Previous experience of augmented reality | Yes | 3 | 6.70 (1.20) | -2.47 | .020 |
|  |  | No | 27 | 8.17 (0.96) |  |  |
| Usefulness | Gender | Female | 19 | 9.24 (0.72) | 1.96 | .060 |
|  |  | Male | 11 | 8.42 (1.59) |  |  |
|  | Previous experience of augmented reality | Yes | 3 | 7.10 (1.67) | -3.37 | .002 |
|  |  | No | 27 | 9.14 (0.93) |  |  |
